# Interactive Effects of Copper Sources and a High Level of Phytase in Phosphorus-Deficient Diets on Growth Performance, Nutrient Digestibility, Tissue Mineral Concentrations, and Plasma Parameters in Nursery Pigs

**DOI:** 10.1007/s12011-021-02580-x

**Published:** 2021-01-11

**Authors:** Ping Ren, Juxing Chen, Deana Hancock, Mercedes Vazquez-Añón

**Affiliations:** Novus International, Inc., St. Charles, MO 63304 USA

**Keywords:** Copper, Fiber digestibility, Growth performance, Phosphorus digestibility, Pigs, Phytase

## Abstract

The present study investigated the interactive effects of copper sources and a high level of phytase on growth performance, nutrient digestibility, tissue mineral concentrations, and plasma parameters in nursery pigs. Weaning piglets (*N* = 192; 6.06 ± 0.99 kg), blocked by body weight, were randomly allotted to 1 of 4 dietary treatments, with 12 pens per treatment and 4 pigs per pen. A basal diet for each phase was formulated to meet nutrient requirements for nursery pigs with the exception that standardized total tract digestibility (STTD) P was reduced by 0.12% and Ca was adjusted to achieve Ca/STTD *P* = 2.15. The 4 dietary treatments were arranged in a 2 × 2 factorial design, with 2 Cu sources (125 mg/kg Cu from copper methionine hydroxy analogue chelate (Cu-MHAC) or copper sulfate (CuSO_4_)) and 2 phytase levels (0 or 1500 phytase units (FTU)/kg). Results showed that there was an interaction (*P* < 0.05) between Cu sources and phytase on ADG during days 0–41. When phytase was not present in the diets (P deficient), there was no difference between the two Cu sources in terms of ADG during days 0–41, whereas with phytase in the diets, Cu-MHAC tended to improve (*P* < 0.10) ADG during days 0–41 compared with CuSO_4_. Pigs fed Cu-MHAC had greater apparent total tract digestibility (ATTD) of neutral and acid detergent fiber and STTD of P than those fed CuSO_4_. Phytase increased (*P* < 0.05) growth performance, ATTD of Ca and P, and plasma inositol and growth hormone concentrations. In conclusion, Cu-MHAC may be more effective in improving growth rate than CuSO_4_ when phytase was supplemented at 1500 FTU/kg. Cu-MHAC enhanced fiber and P digestibility regardless of phytase, compared with CuSO_4_. Phytase addition in P-deficient diets was effective in improving growth performance, Ca and P digestibility, and plasma inositol and growth hormone concentrations.

## Introduction

High levels of copper sulfate (CuSO_4_, 150 to 250 mg/kg Cu) are widely used in nursery pigs to promote growth and improve feed efficiency [1]. It has been demonstrated that chelated Cu is more bioavailable in both chickens and pigs than CuSO_4_ [2, 3]. Supplementation of Cu as Cu-citrate (125 mg/kg Cu) or Cu-proteinate (100 mg/kg Cu) has been shown to achieve similar growth performance in nursery pigs as CuSO_4_ supplementation at 250 mg/kg Cu [4, 5]. Similarly, supplementation of copper methionine hydroxy analogue chelate achieved greater average daily gain and feed efficiency in nursery or grow-finish pigs compared with pigs fed CuSO_4_ or tribasic copper chloride at equivalent Cu levels [6–9].

Copper sulfate is easily dissociated in the acidic pH in the stomach, allowing Cu to interact with phytate and resulting in the formation of Cu-phytate complexes [10, 11]. These complexes could impair phytase efficacy, resulting in lower P release from the phytate molecule [11, 12]. An in vitro model has demonstrated that Cu lysine is less inhibitive to phytase compared with CuSO_4_ [11]. Our recent data also suggests that Cu-MHAC may enhance phytase efficacy on bone mineralization in nursery pigs when 500 FTU/kg phytase is used, compared with CuSO_4_ [9]. However, there is no data on the interaction of copper source and phytase supplementation at high levels on growth performance and mineral digestibility in pigs. It is hypothesized that Cu-MHAC could enhance the efficacy of phytase at 1500 FTU/kg on growth performance and mineral digestibility in nursery pigs, compared with CuSO_4_. Therefore, the objective of this study is to investigate the interactive effects of copper sources and phytase supplementation at 1500 FTU/kg on growth performance, nutrient digestibility, tissue mineral concentrations, and plasma parameters.

## Materials and Methods

### Animals and Management

The present experiment was conducted at Green Acres Animal Research and Testing Facility (Novus International, Inc., Montgomery City, MO). A total of 192 TR4 × C22 weaning barrows (BW 6.06 ± 0.99 kg; PIC, Hendersonville, TN, USA) were used in this study. Pigs were housed in plastic-coated floor pens. Each piglet was tagged for individual identification. Pigs had free access to the feed and water during the entire nursery period. A three-phase feeding program (days 0–14, 15–28, 29–42) was used in the present study.

### Experimental Design and Dietary Treatments

At weaning (day 0), piglets were weighed individually and allotted to 1 of 4 dietary treatments according to a randomized complete block design, which was blocked by initial BW. There were 12 pens per treatment and 4 pigs per pen. A basal diet for each phase was formulated to meet the energy and nutrient requirements for different stages of pigs according to the recommendation by NRC [13], with the exception that standardized total tract digestibility (STTD) P was reduced by 0.12% and Ca level was adjusted to meet the fixed ratio of Ca to STTD P of 2.15. The 4 dietary treatments were arranged in a 2 × 2 factorial design (Table 1), with 2 Cu sources (125 ppm Cu from copper methionine hydroxy analogue chelate (Cu-MHAC, MINTREX® Cu, Novus International, Inc., St. Charles, MO, USA) or CuSO_4_ (Old Bridge Chemicals, Inc., Old Bridge, NJ, USA), respectively) and 2 phytase levels (0 or 1500 FTU/kg phytase (PHYTAVERSE®, Novus International, Inc., St. Charles, MO, USA)). The basal diet composition for the 3 phases is presented in Table 2.

**Table 1:** Description of dietary treatments in terms of copper sources and levels and phytase inclusion levels

Treatment	Cu sources	Cu levels, mg/kg	Phytase^b^ levels
1	Cu-MHAC^a^	125	0
2	CuSO_4_^a^	125	0
3	Cu-MHAC	125	1500
4	CuSO_4_	125	1500

^a^Cu-MHAC represents Cu methionine hydroxy analogue chelate (MINTREX® Cu), which is manufactured by Novus International, Inc., St. Charles, MO, USA. CuSO_4_ is manufactured by Old Bridge Chemicals, Inc., Old Bridge, NJ, USA

^b^Phytase used in this study is a commercial feed-grade phytase (PHYTAVERSE®, Novus International, Inc., St. Charles, MO, USA)

**Table 2:** Ingredient and nutrient composition of basal experimental diets (as-fed basis)

Items	Phase 1 (day 0–14)	Phase 2 (day 15–28)	Phase 3 (day 29–42)
Ingredients, %			
Corn, yellow dent	49.10	54.13	61.45
Soybean meal, 47.5% CP	15.00	20.00	25.00
HP300	4.00	5.00	0.00
Canola meal	4.00	6.00	10.00
Plasma spray-dried	3.00	0.80	0.00
Whey powder	20.00	10.00	0.00
Choice white grease	2.00	2.00	1.50
Dicalcium phosphate 18.5%	0.00	0.00	0.15
Limestone	1.10	0.82	0.63
Salt	0.30	0.30	0.30
Vitamin premix^a^	0.11	0.11	0.11
Mineral premix^b^	0.29	0.29	0.29
Titanium dioxide	0.00	0.00	0.40
ZnO, 72% Zn	0.28	0.00	0.00
L-lysine HCl	0.49	0.36	0.17
MHA^c^	0.19	0.12	0.00
L-Thr	0.13	0.07	0.00
L-Trp	0.01	0.00	0.00
Total	100.00	100.00	100.00
Calculated nutrient composition			
ME, kcal/kg	3455	3437	3367
CP, %	20.24	21.24	20.97
SID^d^ Lys	1.39	1.27	1.06
SID Thr	0.82	0.75	0.64
SID Met	0.43	0.39	0.30
SID Met + Cys	0.76	0.70	0.61
SID Trp	0.24	0.23	0.85
Ca, %	0.62	0.49	0.43
Total P, %	0.49	0.47	0.48
STTD^d^ P, %	0.29	0.23	0.20
Phytate P, %	0.20	0.24	0.31

^a^Supplied the following nutrients per kilogram of diets: vitamin A, 11,000 IU; vitamin D, 1760 IU; vitamin E, 83.6 IU; vitamin K, 5.5 mg; thiamine, 3.52 mg; riboflavin, 13.2 mg; niacin, 70.4 mg; pantothenic acid, 39.6 mg; pyridoxine, 7.04 mg; folic acid, 1045 μg; biotin, 275 μg; vitamin B12, 55 μg

^b^The trace mineral premix supplied the following nutrients per kilogram of diets: Zn, 100 mg as zinc sulfate; Cu, 125 mg as either Cu-MHAC or CuSO_4_ according to Table 1; Mn, 34 mg as manganese sulfate; Fe, 100 mg as ferrous sulfate; I, 0.2 mg as ethylenediamine dihydriodide; Se, 0.3 mg as sodium selenite

^c^MHA® is a dry calcium salt of D, L-2-hydroxy-4-(methylthio)butanoic acid (84% Met activity, MHA®, Novus International, Inc., St. Charles, MO, USA)

^d^*SID* standardized ileal digestible, *STTD* standardized total tract digestible

### Measurements and Sample Collection

Piglet body weights (BW) were measured at the initiation of the study (day 0) and at the end of each phase (days 14, 28, and 41). Feed addition to each feeder was recorded each time when the feed was added to the feeder. At the end of each phase, the remaining feed in the feeder was weighed. Average daily gain (ADG), average daily feed intake (ADFI), and gain-to-feed ratio (G:F) were calculated for each phase and the entire nursery period.

Fresh fecal samples were collected via grab sampling from each pig in each pen twice a day from days 36 to 41. The fecal samples were stored at − 20 °C immediately after collection. At the end of sample collection, fecal samples within each pen were thawed, homogenized, and immediately placed in a heated oven (NHP-PD-ECO, Win-Holt, Woodbury, NY, USA) at 65 °C for 48 h. All dried fecal samples were ground using a rotor mill (PULVERISETTE 14, Fritsch GmbH, Idar-Oberstein, Germany) fitted with a 1-mm screen. Ground feces were thoroughly homogenized, and a subsample was collected for chemical analysis.

At the end of the study (day 42), one pig from each pen with the BW closest to the average BW of the pen was selected and approximately 7 ml of blood samples was collected via jugular puncture, followed by euthanasia using captive bolt. The blood samples were centrifuged at 1500 × g at 4 °C for 15 min to obtain plasma samples. The third metacarpal samples were collected from selected pigs. Liver (approximately 50 g of the right lobe) and kidney (approximately 50 g of the right kidney) samples were collected in the selected pigs. The metacarpal, liver, and kidney samples were stored at − 20 °C until chemical analysis.

### Chemical and Biological Analyses

Diets and feces samples were analyzed for dry matter (DM, method 934.01), Ca (method 985.01), P (method 985.01), Zn (method 985.01), and Cu (method 985.01) according to AOAC [14]. Phytase activity in the diets was analyzed according to ISO [15]. Gross energy was analyzed in diets and feces using an isoperibol bomb calorimeter (Model 6300, Parr Instruments, Moline, IL). Acid detergent fiber (ADF) and neutral detergent fiber (NDF) in diets and feces were analyzed using ANKOM Technology method 12 and 13, respectively (ANKOM 2000 Fiber Analyzer, ANKOM Technology, Macedon, NY). Titanium concentration in phase 3 diets and fecal samples was analyzed according to the procedures described by Myers et al. [16].

Plasma samples were used for malondialdehyde (MDA) analysis, which was quantified using thiobarbituric acid reactive substances assay kit 10009055 (Cayman Chemical Company, Ann Arbor, MI). Plasma inositol was measured using myo-inositol assay kit from Megazyme (Wicklow, Ireland). Growth hormone and insulin-like growth factor-1 (IGF-1) concentrations were measured using porcine growth hormone enzyme-linked immunosorbent assay (ELISA) kit from MyBioSource (San Diego, CA) and porcine IGF-1 ELISA kit, respectively.

Metacarpal, liver, and kidney samples were ashed in a muffle furnace at 600 °C overnight in porcelain crucibles. The concentrations of ash, Ca, P, Zn, and Cu in metacarpal, liver, and kidney samples were determined using the procedure described by method 985.01 of AOAC [14]. Ash, Ca, and P percentages in metacarpal samples were expressed as grams of ash, Ca, and P per 100 g of dry, fat-free metacarpal weight, respectively.

### Calculations

The apparent total tract digestibility (ATTD) coefficient for Ca and P in each treatment was calculated according to equations proposed by NRC [13]. Standardized total tract digestibility of Ca and P were calculated by accounting for endogenous losses of Ca (330 mg/kg dry matter intake) [17] and P (190 mg/kg dry matter intake) [13], respectively.

### Statistical Analysis

SAS® 9.4 (SAS Inst. Inc., Gary, NC) was used for all data analysis. Pen served as the experimental unit. The LSMEANS statement was used to calculate the least square means. The Tukey-Kramer adjustment was used for multiple comparisons of the least square means. Pooled SEM was calculated for each measurement. A probability of *P* ≤ 0.05 was considered as significant and 0.05 < *P* ≤ 0.10 was declared as a trend.

The GLIMMIX procedure was used to analyze all the data. Copper source, phytase level, and their interaction were considered as the fixed effects, whereas block was considered as the random effect. All the data were analyzed using default normal regression model.

## Results

### Effect of Cu Sources and Phytase on Growth Performance

Pigs fed diets containing Cu-MHAC tended (*P* < 0.10) to have greater G:F during days 0–14, compared with those fed diets containing CuSO_4_ (Table 3). Pigs fed diets containing 1500 FTU/kg phytase had greater (*P* < 0.05) ADG, ADFI, and G:F during all phases, except for G:F during days 28–41, compared with those fed diets without phytase.

**Table 3:** Effect of copper sources and phytase supplementation at 1500 FTU/kg on growth performance in nursery pigs^c^

Items	No^d^		Yes^d^		SEM	*P* value	Cu sources		SEM	*P* value	Phytase		SEM	*P* value
	CuSO_4_	Cu-MHAC	CuSO_4_	Cu-MHAC			CuSO_4_^e^	Cu-MHAC^e^			no	yes		
BW, kg														
Day 0	6.1	6.1	6.1	6.1	0.3	0.87	6.1	6.1	0.3	0.41	6.1	6.1	0.3	0.53
Day 14	9.3	9.4	10.1	10.3	0.5	0.71	9.7	9.8	0.5	0.31	9.3^b^	10.2^a^	0.5	< 0.01
Day 28	14.7	14.4	16.7	17.2	0.7	0.10	15.7	15.8	0.7	0.59	14.6^b^	16.9^a^	0.7	< 0.01
Day 41	21.9^b^	21.4^b^	26.0^ay^	27.6^ax^	1.2	0.03	24.0	24.5	1.2	0.27	21.6^b^	26.8^a^	1.2	< 0.01
ADG, g/d														
Days 0 to 14	229	238	284	303	18	0.69	257	270	16	0.26	234^b^	294^a^	16	< 0.01
Day s14 to 28	387	360	469	496	19	0.12	428	428	15	0.97	374^b^	482^a^	15	< 0.01
Days 28 to 41	546^b^	534^b^	704^ay^	790^ax^	46	0.04	625	662	43	0.12	540^b^	747^a^	43	< 0.01
Days 0 to 28	309	299	378	399	15	0.09	343	349	13	0.55	304^b^	388^a^	13	< 0.01
Days 0 to 41	386^b^	373^b^	486^ay^	526^ax^	23	0.03	436	450	21	0.26	380^b^	506^a^	21	< 0.01
ADFI, g/d														
Days 0 to 14	382	369	418	398	23	0.83	400	383	21	0.28	375^b^	408^a^	21	0.04
Days 14 to 28	629	645	665	720	33	0.38	647	683	29	0.11	637^b^	692^a^	29	0.02
Days 28 to 41	805	778	996	1065	48	0.16	901	921	42	0.54	791^b^	1031^a^	42	< 0.01
Days 0 to 28	505	507	541	559	27	0.62	523	533	24	0.56	506^b^	551^a^	24	0.01
Days 0 to 41	597	592	683	718	32	0.32	640	655	28	0.45	595^b^	700^a^	28	< 0.01
G:F, g/g														
Days 0 to 14	0.613	0.650	0.683	0.763	0.037	0.48	0.648	0.707	0.030	0.06	0.631^b^	0.723^a^	0.030	< 0.01
Days 14 to 28	0.618	0.573	0.696	0.692	0.026	0.45	0.657	0.632	0.019	0.35	0.595^b^	0.694^a^	0.019	< 0.01
Days 28 to 41	0.675	0.677	0.696	0.738	0.032	0.38	0.685	0.707	0.028	0.34	0.67^6^	0.717	0.028	0.08
Days 0 to 28	0.613	0.599	0.690	0.716	0.019	0.29	0.652	0.657	0.014	0.76	0.606^b^	0.703^a^	0.014	< 0.01
Days 0 to 41	0.646	0.632	0.695	0.730	0.019	0.13	0.671	0.681	0.015	0.52	0.639^b^	0.713^a^	0.015	< 0.01

^a–b^Within a row, means without a common superscript differ (*P* ≤ 0.05). ^x–y^Within a row, means without a common superscript differ (0.05 < *P* ≤ 0.10)

^c^Least square means for each treatment are calculated based on 12 replicates per treatment and 4 pigs per replicate

^d^No and yes represent 0 and 1500 FTU/kg phytase (PHYTAVERSE®, Novus International, Inc., St. Charles, MO, USA) in the diets, respectively

^e^Cu-MHAC represents Cu methionine hydroxy analogue chelate (MINTREX® Cu), which is manufactured by Novus International, Inc., St. Charles, MO, USA. CuSO_4_ is manufactured by Old Bridge Chemicals, Inc., Old Bridge, NJ, USA

There was an interaction (*P* < 0.05) between Cu sources and phytase in terms of BW on day 41. When phytase was not present in the diets (P deficient), there was no difference between the two Cu sources in terms of BW on day 41. In contrast, when phytase was included in the diets, Cu-MHAC tended to have greater (*P* < 0.10) BW on day 41 than CuSO_4_. Similarly, there was an interaction (*P* < 0.05) between Cu sources and phytase on ADG during days 0–41. When phytase was not present in the diets (P deficient), there was no difference between the two Cu sources in terms of ADG during days 0–41. In contrast, when phytase was included in the diets, Cu-MHAC tended to increase (*P <* 0.10) ADG during days 0–41 compared with CuSO_4_.

### Effect of Cu Sources and Phytase on Nutrient Digestibility

The ATTD of NDF and ADF was greater (*P* < 0.05) in pigs fed diets containing Cu-MHAC compared with pigs fed diets containing CuSO_4_ (Table 4). Pigs fed diets containing Cu-MHAC had greater (*P* < 0.05) ATTD and STTD of P than those fed diets containing CuSO_4_. Phytase supplementation at 1500 FTU/kg increased (*P* < 0.01) ATTD of ash, Ca, and P, and STTD of Ca and P, compared with no phytase supplementation. However, phytase supplementation at 1500 FTU/kg resulted in lower (*P* < 0.05) ATTD of NDF, ADF, and gross energy, compared with no phytase supplementation.

**Table 4:** Effect of copper sources and phytase supplementation at 1500 FTU/kg on total tract digestibility of nutrients in nursery pigs^c^

Items	No^d^		Yes^d^		SEM	*P* value	Cu sources		SEM	*P* value	Phytase		SEM	*P* value
	CuSO_4_	Cu-MHAC	CuSO_4_	Cu-MHAC			CuSO_4_^e^	Cu-MHAC^e^			No	Yes		
ATTD^f^, %														
Ash	56.13	55.94	63.87	66.05	0.99	0.24	60.00	61.00	0.70	0.32	56.04^b^	64.96^a^	0.70	< 0.01
DM	85.78	86.27	85.34	85.99	0.41	0.85	85.56	86.13	0.29	0.18	86.02	85.67	0.29	0.39
EE	68.44	68.42	68.69	69.79	1.01	0.59	68.57	69.10	0.71	0.60	68.43	69.24	0.71	0.43
CF	55.66	53.58	51.45	54.62	1.35	0.03	53.56	54.10	1.07	0.64	54.62	53.03	1.07	0.18
CP	84.45	85.21	83.36	84.03	0.63	0.94	83.91	84.62	0.45	0.27	84.83	83.70	0.45	0.08
NDF	48.60	51.31	41.91	47.94	1.35	0.23	45.26^b^	49.63^a^	0.96	< 0.01	49.96^a^	44.93^b^	0.96	< 0.01
ADF	52.05	54.33	46.26	52.69	1.56	0.19	49.15^b^	53.51^a^	1.10	0.01	53.19^a^	49.48^b^	1.10	0.02
GE	84.63	85.14	83.63	83.73	0.44	0.65	84.13	84.44	0.31	0.49	84.89^a^	83.69^b^	0.31	0.01
Ca	65.92	64.55	79.64	81.12	1.63	0.39	72.78	72.84	1.15	0.97	65.24^b^	80.39^a^	1.15	< 0.01
P	40.07	41.81	65.29	70.32	1.52	0.29	52.68^b^	56.06^a^	1.08	0.03	40.94^b^	67.81^a^	1.08	< 0.01
STTD^f^, %														
Ca	71.79	70.42	85.51	86.99	1.63	0.39	78.65	78.70	1.15	0.97	71.10^b^	86.25^a^	1.15	< 0.01
P	43.44	45.17	68.66	73.68	1.52	0.29	56.05^b^	59.43^a^	1.08	0.03	44.31^b^	71.17^a^	1.08	< 0.01

^a–b^Within a row, means without a common superscript differ (*P* ≤ 0.05)

^c^Total tract digestibility of nutrients is determined based on fecal samples collected from days 36 to 41 post-weaning in nursery pigs. Least square means for each treatment are calculated based on 12 replicates per treatment and 1 pooled fecal sample from 4 pigs per replicate

^d^No and yes represent 0 and 1500 FTU/kg phytase (PHYTAVERSE®, Novus International, Inc., St. Charles, MO, USA) in the diets, respectively

^e^Cu-MHAC represents Cu methionine hydroxy analogue chelate (MINTREX® Cu), which is manufactured by Novus International, Inc., St. Charles, MO, USA. CuSO_4_ is manufactured by Old Bridge Chemicals, Inc., Old Bridge, NJ, USA

^f^*ATTD* apparent total tract digestibility, *DM* dry matter, *EE* ether extract, *CF* crude fiber, *CP* crude protein, *NDF* neutral detergent fiber, *ADF* acid detergent fiber, *GE* gross energy, *STTD* standardized total tract digestibility

### Effect of Cu Source and Phytase on Bone Mineralization

Cu sources did not affect fat-free bone weight, bone ash, Ca, P, Zn, and Cu concentrations from pigs on day 42 (Table 5). Phytase supplementation at 1500 FTU/kg increased (*P* < 0.05) fat-free bone weight and bone P concentrations compared with no phytase supplementation. Similarly, phytase supplementation at 1500 FTU/kg tended to increase (*P* < 0.10) bone ash and Ca concentrations compared with no phytase supplementation. However, phytase supplementation at 1500 FTU/kg reduced (*P* < 0.01) bone Cu concentration compared with no phytase supplementation.

**Table 5:** Effect of copper sources and phytase supplementation at 1500 FTU/kg on metacarpal mineralization in nursery pigs^c^

Items	No^d^		Yes^d^		SEM	*P* value	Cu sources		SEM	*P* value	Phytase		SEM	*P* value
	CuSO_4_	Cu-MHAC	CuSO_4_	Cu-MHAC			CuSO_4_^e^	Cu-MHAC^e^			No	Yes		
Fat-free bone weight, g	2.45	2.69	3.61	3.43	0.43	0.59	3.03	3.06	0.32	0.94	2.57^b^	3.52^a^	0.32	0.02
Bone ash, %	35.55	33.97	41.68	42.36	3.83	0.76	38.62	38.16	2.76	0.90	34.76^y^	42.02^x^	2.76	0.06
Ca, %	13.04	12.53	15.11	15.38	1.41	0.78	14.07	13.96	1.03	0.93	12.79^y^	15.25^x^	1.03	0.08
P, %	6.57	6.28	7.99	8.14	0.73	0.76	7.28	7.21	0.53	0.93	6.42^b^	8.06^a^	0.53	0.03
Cu, mg/kg	2.87	2.52	1.85	1.88	0.25	0.45	2.36	2.20	0.17	0.53	2.69^a^	1.86^b^	0.17	< 0.01
Zn, mg/kg	122.24	129.64	138.49	142.22	13.65	0.89	130.37	135.93	9.94	0.68	125.94	140.35	9.94	0.28

^a–b^Within a row, means without a common superscript differ (*P* ≤ 0.05). ^x–y^Within a row, means without a common superscript differ (0.05 < *P* ≤ 0.10)

^c^Metacarpal samples were collected from nursery pigs on day 42 post-weaning. Least square means for each treatment are calculated based on 12 replicates per treatment and 1 pig per replicate

^d^No and yes represent 0 and 1500 FTU/kg phytase (PHYTAVERSE®, Novus International, Inc., St. Charles, MO, USA) in the diets, respectively

^e^Cu-MHAC represents Cu methionine hydroxy analogue chelate (MINTREX® Cu), which is manufactured by Novus International, Inc., St. Charles, MO, USA. CuSO_4_ is manufactured by Old Bridge Chemicals, Inc., Old Bridge, NJ, USA

### Effect of Cu Source and Phytase on Liver and Kidney Mineral Concentrations

There were no differences (*P* > 0.10) among the two Cu sources in terms of liver and kidney Cu and Zn concentrations (Table 6). Phytase supplementation at 1500 FTU/kg reduced (*P* < 0.01) liver Cu concentration compared with no phytase supplementation. In contrast, phytase supplementation at 1500 FTU/kg resulted in greater (*P* < 0.05) kidney Cu and Zn concentration compared with no phytase supplementation.

**Table 6:** Effect of copper sources and phytase supplementation at 1500 FTU/kg on liver and kidney copper and zinc concentrations in nursery pigs^c^

Items	No^d^		Yes^d^		SEM	*P* value	Cu sources		SEM	*P* value	Phytase		SEM	*P* value
	CuSO_4_	Cu-MHAC	CuSO_4_	Cu-MHAC			CuSO_4_^e^	Cu-MHAC^e^			No	Yes		
Liver														
DM, %	66.07	67.66	71.02	69.16	1.90	0.28	68.55	68.41	1.55	0.93	66.87^b^	70.09^a^	1.55	0.05
Cu, mg/kg	20.76	23.48	17.80	17.00	1.89	0.35	19.28	20.24	1.36	0.61	22.12^a^	17.40^b^	1.37	0.02
Zn, mg/kg	147.30	138.09	127.87	148.46	13.16	0.26	137.59	143.27	9.39	0.67	142.69	138.17	9.39	0.73
Kidney														
DM, %	19.27	18.72	19.65	19.51	0.38	0.57	19.46	19.12	0.28	0.34	19.00	19.58	0.28	0.11
Cu, mg/kg	26.93	28.47	61.62	61.52	6.80	0.90	44.27	44.99	4.93	0.91	27.70^b^	61.57^a^	4.93	< 0.01
Zn, mg/kg	134.15	134.70	168.85	150.65	9.74	0.34	151.50	142.67	6.96	0.37	134.43^b^	159.75^a^	6.95	0.01

^a–b^Within a row, means without a common superscript differ (*P* ≤ 0.05)

^c^Liver and kidney samples were collected from nursery pigs on day 42 post-weaning. Least square means for each treatment are calculated based on 12 replicates per treatment and 1 pig per replicate

^d^No and yes represent 0 and 1500 FTU/kg phytase (PHYTAVERSE®, Novus International, Inc., St. Charles, MO, USA) in the diets, respectively

^e^Cu-MHAC represents Cu methionine hydroxy analogue chelate (MINTREX® Cu), which is manufactured by Novus International, Inc., St. Charles, MO, USA. CuSO_4_ is manufactured by Old Bridge Chemicals, Inc., Old Bridge, NJ, USA

### Effect of Cu Source and Phytase on Plasma Parameters

There were no differences (*P* > 0.10) between the two Cu sources in terms of plasma inositol, TBARS, growth hormone, and IGF-1 concentrations (Table 7). Phytase supplementation at 1500 FTU/kg increased (*P* < 0.05) plasma inositol and growth hormone concentrations compared with no phytase supplementation.

**Table 7:** Effect of copper sources and phytase supplementation at 1500 FTU/kg on plasma parameters in nursery pigs^c^

Items	No^d^		Yes^d^		SEM	*P* value	Cu sources		SEM	*P* value	Phytase		SEM	*P* value
	CuSO_4_	Cu-MHAC	CuSO_4_	Cu-MHAC			CuSO_4_^e^	Cu-MHAC^e^			No	Yes		
Inositol, μg/ml	37.34	40.52	46.30	57.56	5.62	0.45	41.82	49.04	4.16	0.19	38.93^b^	51.93^a^	4.17	0.02
MDA^f^, μM	7.79	7.63	8.75	8.11	0.47	0.58	8.27	7.87	0.36	0.36	7.71	8.43	0.36	0.11
GH^f^, ng/ml	0.12	0.10	0.35	0.25	0.08	0.64	0.23	0.17	0.06	0.45	0.11^b^	0.30^a^	0.06	0.03
IGF-1^f^, ng/ml	205,323	158,680	182,527	185,137	23,136	0.09	193,925	171,908	20,931	0.12	182,002	183,832	20,931	0.90

^a–b^Within a row, means without a common superscript differ (*P* ≤ 0.05)

^c^Blood samples were collected on day 42 post-weaning in the nursery pigs. Least square means for each treatment are calculated based on 12 replicates per treatment and 1 pig per replicate

^d^No and yes represent 0 and 1500 FTU/kg phytase (PHYTAVERSE®, Novus International, Inc., St. Charles, MO, USA) in the diets, respectively

^e^Cu-MHAC represents Cu methionine hydroxy analogue chelate (MINTREX® Cu), which is manufactured by Novus International, Inc., St. Charles, MO, USA. CuSO_4_ is manufactured by Old Bridge Chemicals, Inc., Old Bridge, NJ, USA

^f^MDA represents malondialdehyde concentration; GH represents growth hormone concentration; IGF-1 represents insulin-like growth factor-1 concentration

## Discussion

### Cu Sources and Its Interaction with Phytase on Growth Performance, Nutrient Digestibility, Tissue Mineral Concentrations, and Plasma Parameters

High levels of CuSO_4_ (150 to 250 mg/kg Cu) are traditionally used in weaning pig diets to promote growth and improve feed efficiency [1, 7, 18]. Technology advancement has led to the development of numerous forms of organic Cu, which have been shown to be more bioavailable than CuSO_4_ [3]. Studies have demonstrated that Cu-proteinate [5] and Cu-MHAC [6, 7, 9] achieved greater growth performance in nursery pigs than CuSO_4_ at the same Cu inclusion levels, which was further supported by the current study that Cu-MHAC achieved greater average daily gain than CuSO_4_ in the presence of phytase supplementation at 1500 FTU/kg. The fact that average daily gain in nursery pigs was not different between Cu-MHAC and CuSO_4_ without phytase in the present study indicated that the impact of P deficiency might overshadow the beneficial effect of Cu-MHAC or P deficiency may impair the function and utilization of Cu in the body. Additionally, Cu-citrate yielded similar growth performance as CuSO_4_ in nursery pigs at the same inclusion level of Cu [4]. It was also shown that Cu-MHAC led to greater growth performance in nursery pigs than tribasic copper chloride at the same Cu inclusion levels [8]. These studies suggest that Cu sources play a critical role in its function and utilization in the body, thereby affecting growth performance of pigs.

Several modes of action could be responsible for the growth performance benefits by Cu-MHAC supplementation. Firstly, Cu could exert antibacterial effect directly or indirectly via bile salts and therefore modulate gut microbiota [19–21]. It was reported that supplementation at 100 mg/kg Cu as Cu-methionine or Cu-proteinate increased the proportion of *Lactobacillus* while they reduced the proportion of *Escherichia coli* in the ileal digesta of broilers [22]. Supplementation of 30 mg/kg Cu from Cu-MHAC in broilers decreased the proportion of *Enterobacteriaceae* and *Firmicutes* in the cecum digesta [23] compared with supplementation of 125 mg/kg Cu from CuSO_4_, which indicated that Cu-MHAC could modulate gut microbiota by shifting cecal microbiota to more beneficial microflora. The improved growth rate by Cu-MHAC in the diets containing 1500 FTU/kg phytase in the current study may be derived from the balanced gut microbiota compared with CuSO_4_.

Secondly, Cu supplementation could exert its function via systemic routes. It has been demonstrated that increasing Cu supplementation in weaning pigs could increase serum mitogenic activity, liver superoxide dismutase activity, and pituitary growth hormone mRNA concentrations [20, 24]. It can also elevate serum growth hormone and IGF-1, enhance both ghrelin mRNA expression in the fundic region of the stomach and growth hormone-releasing hormone, and suppress somatostatin mRNA expression levels in the hypothalamus [25–27]. A recent study conducted by Gonzalez-Esquerra et al. [28] demonstrated that nursery pigs fed Cu-MHAC at 50 mg/kg Cu had increased serum growth hormone concentration than those fed CuSO_4_ at 160 mg/kg Cu. However, plasma growth hormone and IGF-1 were not affected by the two Cu sources in the current study. The discrepancy among studies may be attributed to animal source and variation, sampling time, diurnal change of these hormones, and the analysis accuracy of these hormones.

Thirdly, Cu-MHAC, which is composed of one mole of Cu chelated with two moles of DL-2-hydroxy-4-(methylthio)butanoic acid in coordinate covalent bonds, is stable in the upper gastrointestinal tract, which may minimize the formation of Cu-phytate complex and allow more Cu to be absorbed by the epithelial cells in the jejunum and ileum [2, 3, 29]. The reduction of Cu-phytate complexes by Cu-MHAC could also improve P digestibility without phytase supplementation compared with CuSO_4_ [29]. In contrast, approximately 40 to 50% of Cu in CuSO_4_ was formed as insoluble Cu-phytate in the pH range 5.5 to 6.5, which resulted in reduced phytase efficacy to break down the phytate molecule to release P [11]. Indeed, increasing Cu levels as CuSO_4_ in the diets containing 600 FTU/kg resulted in a linear reduction of average daily gain, feed efficiency, and apparent P retention rate in broilers [12], which reinforced that CuSO_4_ at high inclusion levels could impair phytase efficacy. However, the in vitro model demonstrated that the magnitude of inhibition on phytate hydrolysis by phytase was much less when Cu-lysine was used as Cu source compared with CuSO_4_ [11], most likely indicating that a lower level of Cu-phytate complex was formed when using chelated Cu source. To the best of our knowledge, the current study was the first study to report increased P digestibility by chelated Cu compared with CuSO_4_ in pigs in the presence of phytase. It was not known why the improved P digestibility by Cu-MHAC did not lead to increased bone P compared with CuSO_4_, which warrants further investigation. The improved ADF and NDF digestibility by Cu-MHAC supplementation compared with CuSO_4_ could also partially explain the improved growth rate in nursery pigs in this study. It has been reported that Cu supplementation at 10–20 mg/kg could increase NDF digestibility in cashmere goats probably due to enhanced rumen fermentation [30]. It was probable that Cu-MHAC supplementation could increase the number of fiber-degrading gut microbes in pigs, thereby improving fiber digestibility, compared with CuSO_4._

### Phytase on Growth Performance, Nutrient Digestibility, Tissue Mineral Concentrations, and Plasma Parameters

The observation that phytase increased growth performance and bone mineralization in pigs was also observed in previous experiments [31–33]. The improved growth performance and bone mineralization by phytase supplementation were mainly a result of increased P digestibility [33–35], which was consistent with the findings of the current study.

Phytate is negatively charged in aqueous solutions, especially under acidic conditions, which makes the phytate molecule bind other nutrients, including starch, protein, and minerals [36]. Additionally, phytate may bind to endogenous digestive enzymes and thereby reduce their capacity to digest nutrients [37]. As a result, increasing phytate levels in the diets may reduce pepsin activity in weanling pigs [38] and intestinal α-amylase, sucrase, and maltase activities in chickens [39]. Phytase supplementation may result in a step-wise hydrolysis of the phytate molecule, thereby releasing phytate bound P and other nutrients, which may improve the digestibility of these nutrients.

Results of experiments to study phytase supplementation on the ATTD of nutrients and energy have been inconsistent. Increasing the dosage of phytase from 0 to 4000 FTU/kg did not affect the ATTD of dry matter and gross energy in growing pigs fed corn-soybean meal–based diets [40], but a linear increase in the ATTD of dry matter and gross energy was observed if the dosage of phytase was increased in corn-soybean meal diets fed to nursery pigs [41]. Increasing levels of phytase from 0 to 4000 FTU/kg did not affect the ATTD of NDF, but linearly increased the ATTD of ADF by nursery pigs fed corn-soybean meal diets [40]. However, a linear reduction in the ATTD of NDF was observed when phytase supplementation was increased from 0 to 20,000 FTU/kg in nursery pigs fed a corn-soybean meal diet [42], which was consistent with the findings of the current study. Phytase in the current study could reduce Ca and P flow to the hindgut, thereby impairing the microbial fermentation and reducing fiber digestibility [43]. The reduced fiber digestibility may partially explain the reduced energy digestibility by phytase in the present study.

It is well documented that phytase supplementation could increase Zn digestibility and bioavailability in pigs [9, 44–46]. However, phytase effect on Cu bioavailability in pigs was inconsistent. Some studies reported phytase supplementation improved Cu digestibility and bioavailability [34, 46], whereas other studies showed no response or even negative effect on Cu digestibility and bioavailability in pigs [45–48]. The amount of Zn release from the phytate complex by phytase supplementation and relative Zn and Cu concentrations in the diets might explain the various responses from different studies. Hill et al. [49] reported the antagonistic effect of excess dietary Zn on Cu availability in pigs. The antagonism between Zn and Cu is primarily mediated through the absorption process, where Zn induces high concentrations of metallothionein in the intestinal mucosa and the binding affinity of metallothionein for Cu is greater than Zn [50, 51]. The Cu bound to metallothionein is not absorbed and sloughed with mucosal cells, therefore reducing Cu bioavailability [52]. To our best knowledge, it is the first study to report the increased kidney Cu concentrations caused by phytase supplementation, along with the reduced bone and liver Cu concentrations. It seems that the improved Zn bioavailability caused by phytase supplementation at 1500 FTU/kg not only impacts Cu absorption in the intestinal level but also shifts Cu metabolism and distribution among various tissues, including bone, liver, and kidney. Further studies are warranted to explore this intriguing question.

Numerous studies have reported that phytase supplementation above 1000 FTU/kg could lead to increased plasma inositol concentration in pigs and broilers [53–55]. It has been suggested that inositol could stimulate insulin signaling pathway and protein synthesis [56], which may explain the improved growth performance of phytase supplementation above 1000 FTU/kg in pigs and broilers [54, 57]. Plasma growth hormone concentration was reduced under phosphorus deficiency in broilers [58, 59]. It could be speculated that increased P bioavailability by phytase supplementation at 1500 FTU/kg in the current study may contribute to enhanced pituitary activity and thereby higher growth hormone secretion. The increased plasma inositol and growth hormone may partially explain the improved growth performance by phytase in the present study.

In conclusion, Cu-MHAC led to greater growth rate in nursery pigs than CuSO_4_ in the presence of phytase supplementation at 1500 FTU/kg. Cu-MHAC enhanced fiber and P digestibility regardless of phytase in comparison with CuSO_4_. Phytase addition to P-deficient diets was effective in improving growth performance, Ca and P digestibility, and plasma inositol and growth hormone concentrations.

## Data Availability

The datasets generated during and/or analyzed during the current study are available from the corresponding author on reasonable request.
